# Temperance Societies

**Published:** 1871-11

**Authors:** 


### TEMPERANCE SOCIETIES.

In the many millions of pages printed on the Temperance question, the best solution of it has not yet been reached; but there has been a gradual approximation to just and true views. The friends of the cause have injured it greatly by contending for too much, and by their exaggerations, in order to make out a strong and striking case; and eminent medical men have allowed themselves to be drawn into the current of false philosophies on the subject. Truth is elicited by degrees on all questions, and best, by the calm expression of the sentiments of individuals. It has always been contended in the pages of Hall's Journal of Health, that as to safety in the use of spirituous liquors, there was only one indisputably true course: He who never tastes a single drop of liquor will never die in the gutter. Whoever does take a single drop, may.

A number of our eminent men seem to have arrived at the following conclusions: that —

Alcohol, in various forms, is a valuable medicine and beverage.

There were two kinds of wine, "old" and "new," mentioned in the Sacred Scriptures, differing only in the amount of alcohol contained, proportioned to age; the use of both was practiced by good men, and inculcated by inspired teachings. "The old is better" for the purposes of wine.

The temperate use of spirituous liquors is the right of all, and in itself is not sinful, nor contrary to Bible authorization; they are beneficial in various conditions of the human system, make an agreeable beverage for the feeble and the old, nor is there anything which can be substituted for them as well.

The societies hitherto organized for the prevention of intemperance were founded on wrong principles, and can never accomplish the ends intended.

The laws heretofore enacted for the total prohibition of the sale of liquor are inefficient, and tend to deprave public morals by offering pecuniary temptation to their violation, or evasion by smuggling.

It is a great risk to cure a person of disease by the employment of any form of spirituous liquors, as it may, and often does engender a taste and habit of use, which not one in many thousands has force of character enough to abandon. It is better to die of disease, than to die of drunkenness. But as the habit of drunkenness does not necessarily follow the employment of ardent spirits for the cure of any malady, their use, where applicable, and where the prospect of their efficiency is more inviting than from any other remedy, under all the circumstances of the case, is warrantable.

While we have the right to use liquors as an occasional beverage and a medicine, and such use is not sinful, yet a generous, noble, and exalted Christian principle points to their total rejection as an habitual beverage, on the same principle that Paul abstained from eating meat, because some professing Christians had been educated to look on meat offered to idols as a blasphemy, and could not see how any man could be a Christian and eat such meat. And yet Paul had a right to eat meat, and would have committed no sin in doing so; on the same ground, men have the right to use spirituous liquors, that is alcohol, in any form, and without sin, as an occasional beverage, for the stomach's sake, and for bodily infirmity, bodily debility.

Signing temperance pledges can never banish intemperance from the land; nor can it ever be done, except by the general prevalence of a high moral sentiment.

The banishment of artificial stimulants from any large community of persons need never be expected; all that we need hope for, this side heaven, is the control of their use within moderate bounds. Among intelligent communities such control, or restriction, will never result from prohibitory laws; the object would more probably result from the following enactments: —

1. Those only to sell liquor who pay a liberal license.

2. No person to be licensed without the consent of a majority of the legal voters within specified boundaries.

3. No one to be licensed who does not own unincumbered real estate.

4. Those who are licensed to be held pecuniarily responsible for any loss of life, or limb, or property, clearly traceable to the liquor sold on their premises.

